# Supplementary material for: AffyMAPSDetector: a software tool to characterize Affymetrix GeneChip™ expression arrays with respect to SNPs
Source: BMC Bioinformatics. 2007 Jul 30;8:276. doi: 10.1186/1471-2105-8-276 (PMC1959249; doi:10.1186/1471-2105-8-276)
Supplement: Additional file 13 — AffyMAPSDetector v1 source code. AffyMAPSDetector source code. [file 1471-2105-8-276-S13.zip › AffyMAPSDetector_v1_src/doc/jwf/package-frame.html]

jwf


jwf

Interfaces 
  
*WizardListener*

Classes 
  
ErrorMessageBox
  
NullWizardPanel
  
Wizard
  
WizardAdapter
  
WizardContext
  
WizardPanel
  
WizardUtils
